# Genetic findings in Parkinson's disease and translation into treatment: a leading role for mitochondria?

**DOI:** 10.1111/j.1601-183X.2007.00342.x

**Published:** 2008-03

**Authors:** V Bogaerts, J Theuns, C van Broeckhoven

**Affiliations:** † Neurodegenerative Brain Diseases Group, Department of Molecular Genetics, VIB Antwerpen, Belgium; ‡ Laboratory of Neurogenetics, Institute Born-Bunge Antwerpen, Belgium; § University of Antwerp Antwerpen, Belgium

**Keywords:** Genetics, Mitochondria, Neuroprotection, Nuclear-encoded proteins, Oxidative stress, Parkinson's disease, Treatment

## Abstract

Parkinson’s disease (PD) is a progressive neurodegenerative movement disorder and in most patients its aetiology remains unknown. Molecular genetic studies in familial forms of the disease identified key proteins involved in PD pathogenesis, and support a major role for mitochondrial dysfunction, which is also of significant importance to the common sporadic forms of PD. While current treatments temporarily alleviate symptoms, they do not halt disease progression. Drugs that target the underlying pathways to PD pathogenesis, including mitochondrial dysfunction, therefore hold great promise for neuroprotection in PD. Here we summarize how the proteins identified through genetic research (*α-synuclein*, *parkin*, *PINK1*, *DJ-1*, *LRRK2* and *HTRA2*) fit into and add to our current understanding of the role of mitochondrial dysfunction in PD. We highlight how these genetic findings provided us with suitable animal models and critically review how the gained insights will contribute to better therapies for PD.

Parkinson’s disease (PD) is the most common neurodegenerative movement disorder with a prevalence of 1.8% in individuals of 65 years and older (de Rijk *et al.* 2000). Resting tremor, bradykinesia and rigidity are the cardinal clinical characteristics of PD. Neuropathological examination shows several affected brain regions, but the loss of dopaminergic (DAergic) neurons in the substantia nigra pars compacta (SNpc) is believed to be the most crucial (Braak *et al.* 2004). At the time of clinical presentation approximately 50–70% of DAergic neurons in the nigrostriatal system have been lost (Orth & Schapira 2002). Surviving neurons may contain Lewy bodies, intracytoplasmic protein aggregates mainly composed of α-synuclein (SNCA) (Spillantini *et al.* 1997). And these Lewy bodies are a second neuropathological feature of PD. Current treatments for PD, with levodopa as the most commonly used drug, are focused on the symptomatic improvement of motor features related to the above mentioned loss of DAergic neurons (Schapira 2005). More importantly levodopa, notwithstanding its symptomatic benefits, does not cure PD, nor does it halt the development of additional features during the course of PD, such as autonomic dysfunction, gait disturbance, freezing and dementia (Olanow *et al.* 2004).

Mitochondrial dysfunction has long been implicated in PD pathogenesis; this hypothesis arose with the discovery that 1-methyl-4-phenyl-1,2,3,6-tetrahydropyridine (MPTP) produced PD-like symptoms in designer drug abusers (Langston *et al.* 1983). Its metabolite, 1-methyl-4-phenylpyridinium (MPP^+^), is actively transported into DAergic neurons by the dopamine transporter. Within these neurons MPP^+^ enters mitochondria, and selectively inhibits mitochondrial respiration at complex I of the electron transport chain (Krueger *et al.* 1990). Chronic infusion of rotenone, a highly selective complex I inhibitor, also reproduced behavioural (e.g. hypokinesia and rigidity) and neuropathological features of PD in rats (Betarbet *et al.* 2000; Sherer *et al.* 2003). These neurotoxins and neurotoxic animal models of PD renewed interest in possible environmental causes of PD, as similar compounds in the environment might play a causative role in the disease. In addition, genetic defects causing familial forms of PD have been identified in the last decade. Despite the rarity of these familial forms of PD (5–10% of the PD population) the identification of PD-linked genes has fuelled our understanding of possible pathogenic mechanisms of PD, and placed ubiquitin-proteasome system (UPS) dysfunction, oxidative stress and mitochondrial dysfunction at centre stage. Mutations or polymorphisms in both mitochondrial DNA (mtDNA) and nuclear DNA were implicated in causing PD or in affecting PD risk. Of the nuclear genes, mutations in *SNCA*, *PARK2* (also known and hereafter referred to as *parkin*), *PINK1* (*PTEN induced putative kinase 1*), *PARK7* (also known and hereafter referred to as *DJ-1*), *LRRK2* (*leucine-rich repeat kinase 2*) and *HTRA2* (*high temperature requirement A2*) provide direct or indirect evidence for a major role of mitochondrial dysfunction in PD.

In this review we aim to highlight the recent genetic data on mtDNA polymorphisms and the nuclear-encoded *SNCA*, *parkin*, *PINK1*, *DJ-1*, *LRRK2* and *HTRA2*, and focus on the relevance of these genes for mitochondrial function. Pathogenic theories focus on a combination of genetic and environmental risk factors, and thus we will also briefly consider environmental exposures relevant to mitochondrial dysfunction in PD. Our current understanding of the mitochondrial pathway to PD provided us with tools to create better animal models, as well as interesting entry points for therapy. These entry points will be discussed along with therapeutic drugs acting on them. Some of the drugs are already in clinical use; others are still in the primary stages of evaluation, but show great promise for modifying PD progression.

## Mitochondria

### Function and structure

The primary function of mitochondria is the generation of cellular energy in the form of adenosine 5′-triphosphate (ATP) by oxidative phosphorylation. In addition to energy production mitochondria play a role in the metabolism of e.g. amino acids and lipids, as well as intermediate metabolic pathways, calcium homeostasis and free radical scavenging.

**Figure 1:**
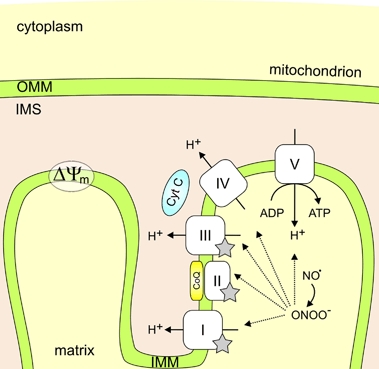
**Mitochondrial structure and composition of the mitochondrial respiratory chain.** The mitochondrial respiratory chain is a sequence of complexes found in the IMM that accepts electrons from electron donors such as NADH or succinate, shuttles these electrons across the IMM and creates a proton and electrochemical gradient. This gradient forms the basis of the inner mitochondrial transmembrane potential (ΔΨ_m_) and is used to drive ATP synthesis by complex V of the respiratory chain. Complexes I, II and III generate ROS (indicated by grey stars). NO inhibits respiration by reversible binding to the oxygen binding site of complex IV (not shown) and is likely to be a physiological regulator of respiration. When cells are under oxidative stress, ROS will accumulate, react with NO, and form peroxynitrite (ONOO^−^); a strong oxidant thought to be responsible for the ‘pathological actions’ of NO. ONOO^−^ inactivates the respiratory complexes (dotted lines), stimulates proton leakage through the IMM and might inhibit complex I by tyrosine nitration (for review see Brown & Borutaite 2002). Abbreviations: ΔΨ_m_, inner mitochondrial transmembrane potential; ADP, adenosine 5′-diphosphate; CoQ, coenzyme Q; Cyt C, cytochrome *c*.

Mitochondria are intracellular double membrane-bound structures (Fig. 1), and are partitioned in four main compartments: outer mitochondrial membrane (OMM), intermembrane space (IMS), inner mitochondrial membrane (IMM) and matrix. The five complexes – I [NADH (nicotinamide adenine dinucleotide, reduced form) ubiquinone oxidoreductase], II (succinate ubiquinone oxidoreductase), III (ubiquinone-cytochrome *c* reductase), IV (cytochrome oxidase) and V (ATP synthase) – of the mitochondrial respiratory chain, also called the electron transport chain, are all located in the IMM. Two mobile electron carriers, coenzyme Q (ubiquinone) and cytochrome *c*, are located in the IMM and IMS, respectively. The transport of electrons down the respiratory chain is energetically favourable. The released energy is used by complexes I, III and IV to transport protons from the matrix to the IMS, thus creating a proton and electrochemical gradient across the IMM. This gradient forms the basis of the inner mitochondrial transmembrane potential (ΔΨ_m_) and is exploited by complex V of the respiratory chain to drive ATP synthesis.

The mitochondria contain their own genome. mtDNA is a multicopy, maternally inherited, 16.5-kb circular double stranded DNA molecule without any histone coating. mtDNA is extremely compact (93% coding sequence) and the majority of proteins required to build and maintain functional mitochondria is therefore encoded by nuclear DNA, synthesized in the cytosol and imported into mitochondria, where they are targeted to one of the four mitochondrial compartments.

### Oxidative stress

**Figure 2:**
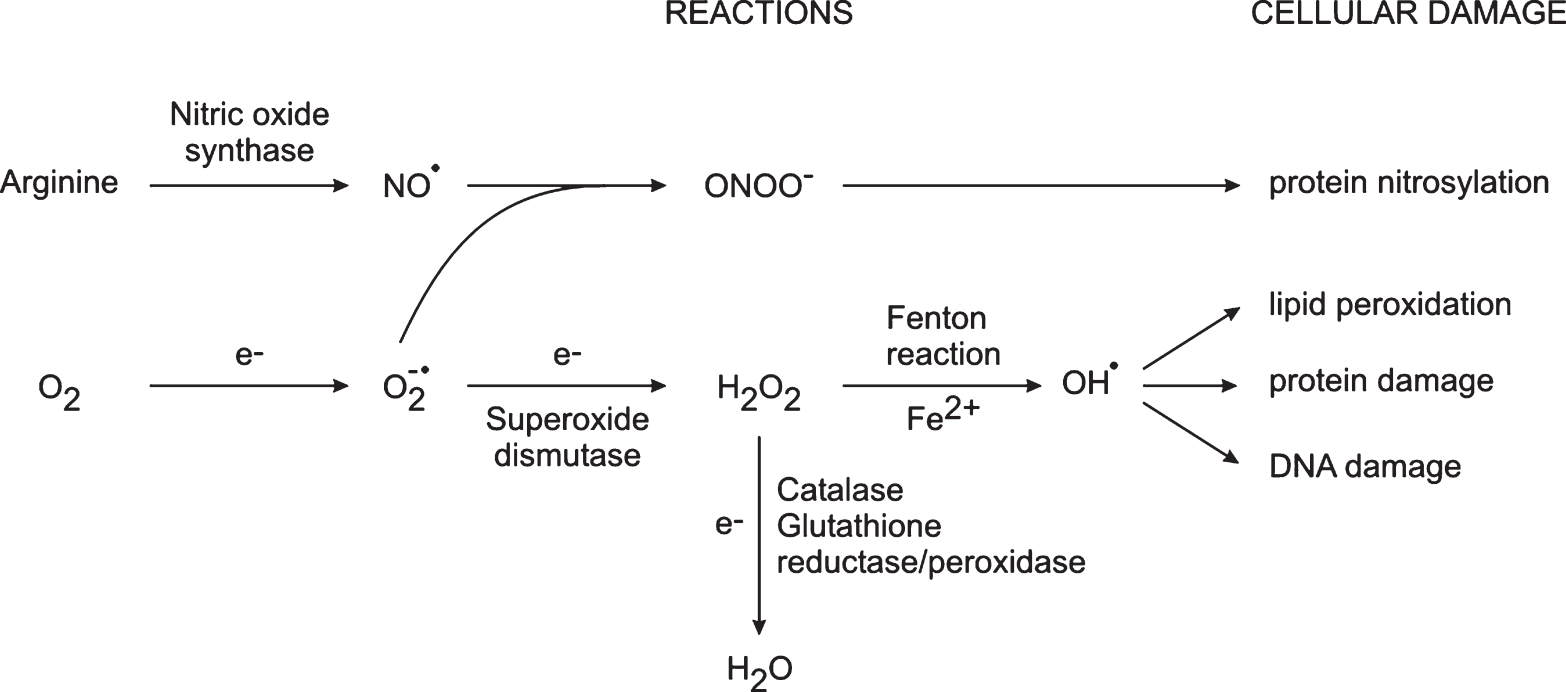
**ROS.** These include (1) free radicals (containing highly reactive unpaired electrons), such as superoxide (O_2_^−·^), nitric oxide (NO^·^) and hydroxyl radical (OH^·^); and (2) other molecular species, such as hydrogen peroxide (H_2_O_2_) and peroxynitrite (ONOO^−^). O_2_^−·^ is converted to H_2_O_2_, either spontaneously or through a reaction catalysed by SOD (Fridovich 1995). H_2_O_2_ may in turn be fully reduced to water, by catalase or glutathione reductase/peroxidase, or partially reduced to hydroxyl radicals (OH^·^). The latter reaction (Fenton reaction) occurs in the presence of reduced transition metals (e.g. Fe^2+^), which may again be re-reduced by O_2_^−·^, propagating the process (Liochev & Fridovich 1994). Alternatively O_2_^−·^ can also react with NO radicals (NO^·^; produced by nitric oxide synthase during conversion of arginine to citrulline) to form peroxynitrite (ONOO^−^) (Beckman & Koppenol 1996).

Oxidative stress is the result of an imbalance between excessive production of reactive oxygen species (ROS) and limited antioxidant defences. Mitochondria generate most of the ROS as a byproduct of oxidative phosphorylation. An estimated 2% of the oxygen (O_2_) consumed by mitochondria is converted to superoxide anion (O_2_^−·^) (Beal 2003), which is a precursor of most other ROS (Turrens 2003) (Fig. 2). These ROS can lead to oxidative damage of proteins, DNA and lipids (Raha & Robinson 2000).

Numerous studies indicated the involvement of ROS and oxidative stress in PD pathogenesis, including reduced amounts of the thiol-reducing agent glutathione (Pearce *et al.* 1997) and elevated concentrations of iron (Fe) (Kienzl *et al.* 1995) in SN of PD patients. Loss of neuromelanin-containing DAergic cells is characteristic for PD and the dark brown pigment neuromelanin attracted attention to the auto-oxidation of dopamine, as it consists primarily of products of dopamine redox chemistry (Wakamatsu *et al.* 2003). Normal metabolism of dopamine, partly accomplished by monoamine oxidases (MAO), produces hydrogen peroxide (H_2_O_2_) (Maker *et al.* 1981). From this reaction alone, DAergic neurons are exposed to oxidative stress. In addition, dopamine can be oxidized to a dopamine quinone. This oxidation occurs spontaneously, is accelerated by the presence of transition metal ions, or can be enzyme-catalysed. The resulting dopamine quinone covalently modifies cellular macromolecules, which may serve as a mechanism for dopamine induced neurotoxicity (Stokes *et al.* 1999).

### Apoptosis

**Figure 3:**
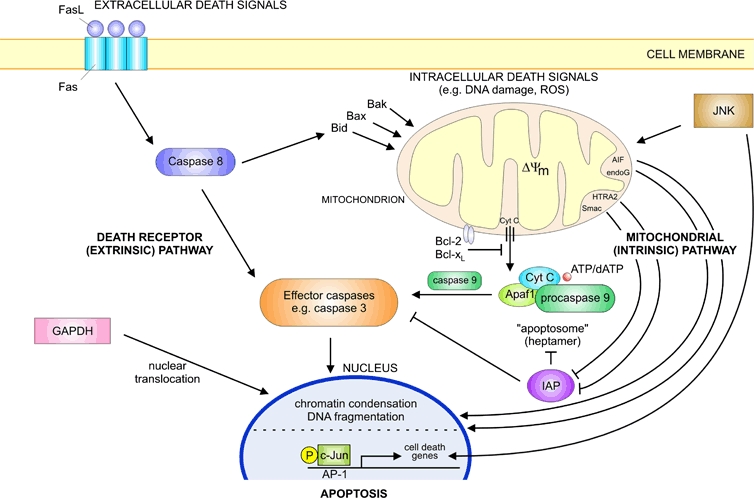
**Schematic illustration of the major pathways leading to apoptosis.** Apoptosis occurs through two main pathways. These are the death receptor (extrinsic) pathway which originates through the activation of cell-surface death receptors, for example Fas, and the mitochondrial (intrinsic) pathway which originates from mitochondrial release of cytochrome *c*. A distinct nuclear pathway of apoptosis arises through increased expression of GAPDH and its translocation from the cytoplasm to the nucleus, whereas the JNK signal-transduction pathway is activated in response to different stress stimuli. Bid, Bax and Bak represent pro-apoptotic Bcl-2 family members; Bcl-2 and Bcl-x_L_ are antiapoptotic. Abbreviations: ΔΨ_m_, inner mitochondrial transmembrane potential; AP-1, activating protein-1; Apaf1, apoptotic protease-activating factor 1; dATP, deoxyadenosine 5′-triphosphate; Cyt C, cytochrome *c*; endoG, endonuclease G; FasL, Fas ligand.

Apoptotic cell death is characterized by marked nuclear and cellular shrinkage, membrane blebbing, chromatin condensation, nuclear fragmentation and the budding off of apoptotic bodies (Kerr *et al.* 1972). Apoptosis is triggered by a number of insults including e.g. misfolded proteins, ROS and mitochondrial complex inhibition (Bredesen *et al.* 2006), and is executed via two main pathways (Fig. 3), which eventually converge at the level of effector caspases activation and the subsequent cleavage of apoptotic substrates. Firstly, the death receptor (or extrinsic) pathway, which is initiated by activation of cell-surface death receptors (e.g. Fas), and secondly, the mitochondrial (or intrinsic) pathway, characterized by the release of mitochondrial pro-apoptotic proteins (e.g. cytochrome *c*) (Hengartner 2000).

The pivotal event in the mitochondrial pathway is mitochondrial outer membrane permeabilization (MOMP) that leads to the release of several IMS proteins, such as cytochrome *c*, apoptosis inducing factor (AIF), endonuclease G, second mitochondria-derived activator of caspases (Smac) and HTRA2 (van Loo *et al.* 2002). MOMP can occur via two mechanisms: the first one involves the opening of the permeability transition (PT) pore, a protein complex at the contact site between OMM and IMM (Zamzami & Kroemer 2001), whereas the second mechanism appears to be mediated by direct action of Bcl-2 family members on the OMM (Green & Kroemer 2004). Cytochrome *c*, released from mitochondria following MOMP, assists in the formation of the apoptosome (a complex consisting of cytochrome *c*, apoptosis protease-activating factor 1, procaspase 9 and ATP/deoxyATP). Apoptosome oligomerization activates caspase 9, which then triggers activation of caspase 3 and other caspases in an amplication cascade ultimately causing cell death (Li *et al.* 1997).

Inhibitors of apoptosis (IAPs) can still inhibit active caspases (Holley *et al.* 2002), but the IAP-mediated block may in turn be released by proteins as Smac (Du *et al.* 2000) or HTRA2 (Martins *et al.* 2002). Two other proteins released from mitochondrial IMS during MOMP (AIF and endonuclease G) translocate to the nucleus and induce chromatin condensation and DNA fragmentation, independent of caspase activation (Li *et al.* 2001; Susin *et al.* 1999).

The c-Jun N-terminal kinase (JNK) signal-transduction pathway is also important for the execution of apoptosis in response to different stress stimuli (Davis 2000). JNK is part of a sequential kinase-signalling cascade involving three kinases. Mitogen-activated protein kinase (MAPK) kinase activates JNK, whereas MAPK kinase activation is mediated by MAPK kinase kinases, including the mixed lineage kinase (MLK) family in neurons. The activation of the JNK pathway rapidly induces its downstream target activating protein-1 transcription factor c-Jun, which plays a major role in the transcription of several pro-apoptotic genes (Ham *et al.* 2000). JNK acts as an effector of apoptosis through mitochondrial-dependent processes, as it catalyses the phosphorylation of antiapoptotic (Schroeter *et al.* 2003) and pro-apoptotic (Bhakar *et al.* 2003; Papadakis *et al.* 2006) Bcl-2 family members, induces release of cytochrome *c* (Schroeter *et al.* 2003) and Smac (Chauhan *et al.* 2003) and mediates a partial collapse of the mitochondrial membrane potential (Schroeter *et al.* 2003). Another distinct pathway of apoptosis arises through increased expression of glyceraldehyde-3-phosphate dehydrogenase (GAPDH) and its translocation from the cytoplasm to the nucleus (Ishitani *et al.* 1996; Sawa *et al.* 1997). In this pathway nitric oxide (NO) is the initial trigger to cell death (Hara *et al.* 2005).

Although an increased immunoreactivity for effectors of the mitochondrial apoptotic pathway (e.g. caspase 3) (Tatton 2000), an increased activation of JNK downstream targets (Hunot *et al.* 2004), as well as nuclear accumulation of GAPDH (Tatton 2000) have been detected in post-mortem SN of PD patients, there is still no clear consensus concerning the contribution of apoptosis to loss of DAergic neurons in PD (Tatton *et al.* 2003b). Necrosis (or passive cell death) represents a form of non-apoptotic cell death, but is not likely to play a major role in PD (Kostrzewa 2000) because necrosis is associated with energy failure, cell swelling and rupture followed by an inflammatory response; features that are largely absent in PD (Dickson 2007). Another form of non-apoptotic cell death that has gained interest in recent years is autophagic cell death, resulting from excessive levels of cellular autophagy. Autophagy complements the UPS pathway as it degrades long-lived proteins, protein aggregates and organelles (e.g. damaged mitochondria) through a lysosomal degradation pathway (Rubinsztein 2006). Whereas the survival-promoting role of autophagy in nutrient starvation is well accepted, its role in cell death is more controversial (Eskelinen 2005). Cross-talk between apoptotic and autophagic pathways has been reported, but their molecular interdependence is not yet clear (Ferraro & Cecconi 2007). However, mitochondria might represent the link at which apoptosis and autophagy interact, because mitochondria generate apoptotic signals, but are in turn removed by autophagy when damaged.

## Environmental exposures and PD

The finding that humans intoxicated with MPTP develop a syndrome nearly identical to PD (Langston *et al.* 1983) lended support to the hypothesis that substances in the environment might contribute to PD aetiology. Indeed, epidemiological studies have implicated environmental risk factors such as rural living, consumption of well water and pesticide exposure in increased risk of PD (Priyadarshi *et al.* 2001). Conversely, cigarette smoking and coffee drinking are inversely associated with the risk for developing PD (Hancock *et al.* 2007; Hernan *et al.* 2002).

Cigarette smoking represents a good example of the relevance of an environmental toxin to mitochondrial function. Cigarette smoke is composed of more than 4000 compounds, but nicotine, the main alkaloid, seems to be particularly important. Nicotine reduces ROS generation from mitochondria (Cormier *et al.* 2003), and prevented neurotoxin-induced mitochondrial swelling and cytochrome *c* release *in vitro* through inhibition of the mitochondrial PT pore (Xie *et al.* 2005). The antioxidant properties of nicotine are likely important as well; e.g. nicotine may be protective against PD through complex formation with Fe^2+^, thus yielding Fenton-inactive Fe^2+^ (Fig. 2) and less oxidative stress (Linert *et al.* 1999). Chronic nicotine treatment was already shown to reduce paraquat-mediated nigrostriatal damage in a rodent model (Khwaja *et al.* 2007).

## Genetics of PD

### mtDNA genetics and PD

Studies using PD cybrids (cytoplasmic hybrids, through transfer of mtDNA of PD patients into mtDNA depleted cells) showed loss of complex I activity, increased O_2_ radical production, and increased susceptibility to MPTP-induced cell death (Swerdlow *et al.* 1996), suggesting that mtDNA abnormalities may be crucial in the pathogenesis of sporadic PD. Several studies reported inherited mtDNA microdeletions or single nucleotide mutations resulting in parkinsonism, typically as one feature of a multisystemic disorder, e.g. the prominent parkinsonism associated with Leber’s hereditary optic neuropathy caused by a single nucleotide mutation in the complex I *ND4* gene (Simon *et al.* 1999). There is, however, less evidence for mtDNA involvement in non-syndromic PD. Nevertheless, specific mtDNA haplotypes were proposed to be implicated in PD risk (Autere *et al.* 2004; Ghezzi *et al.* 2005; Pyle *et al.* 2005; van der Walt *et al.* 2003). Interestingly, recent research into the extent of mtDNA deletions in SN DAergic neurons showed increased levels of clonally expanded somatic mtDNA deletions with ageing (Bender *et al.* 2006b; Kraytsberg *et al.* 2006), resulting in loss of mitochondrial function and cell death. The accumulation of mtDNA deletions was higher in PD patients as compared with age matched control individuals (Bender *et al.* 2006b).

### PD genetics of nuclear-encoded proteins

Linkage analysis in extended families with highly penetrant Mendelian forms of PD has been successful in identifying specific disease-segregating mutations in previously unknown genes implicated in PD pathogenesis. Mutations in at least six genes were shown to cause familial parkinsonism: mutations in *SNCA* and *LRRK2* account for autosomal dominant forms of PD, whereas mutations in *parkin*, *PINK1*, *DJ-1* and *ATP13A2* (Ramirez *et al.* 2006) show a recessive mode of inheritance (mutations in the latter are characteristic of Kufor-Rakeb syndrome, and will not be further discussed). In general autosomal dominant inherited mutations are gain-of-function mutations, while loss-of-function mutations are usually linked to recessive phenotypes. The use of animal models to evaluate gain- and loss-of-function mutations helped greatly in the illumination of the biological functions of the proteins encoded by these genes and yielded insights into their causal role in PD pathogenesis. Transgenic mice provide the ideal means to study possible disease-related gain-of-function mutations, knockout strategies in turn provide the opportunity to study diseases related to loss-of-function mutations.

**Figure 4:**
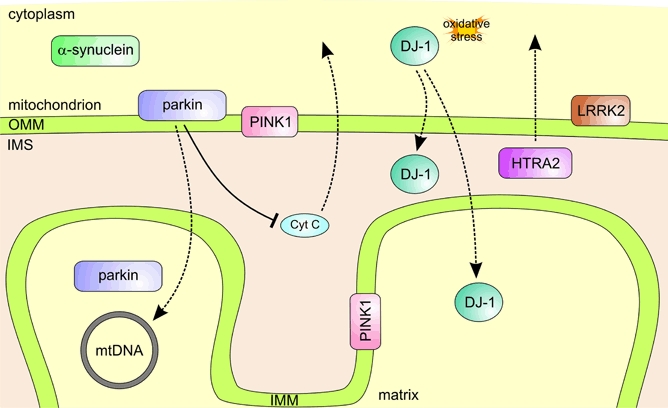
**PD genes and their relation to mitochondria.** Aggregation of mutant or overexpressed SNCA might be an upstream actor of mitochondrial alterations. Parkin associates with the OMM and was shown to play a role in mitochondrial biogenesis by regulating both transcription and replication of mtDNA. PINK1 has an N-terminal mitochondrial targeting motif and is localized to mitochondrial membranes, whereas oxidation of a key Cys-residue in DJ-1 leads to its relocalization to mitochondria. LRRK2 resides diffusely throughout the cytosol, but is partly associated with the OMM. HTRA2 resides in the IMS, wherefrom it is released upon apoptotic stimuli.

The role of Mendelian genes in the common sporadic forms of PD is less known. On the other hand, association studies of candidate genes for PD try to define risk alleles that contribute to the sporadic forms of disease. Candidate gene studies evaluate genes based on their location in previously determined linkage regions (positional candidate genes), or based on a plausible biological function related to disease pathogenesis (functional candidate genes). Table 1 provides a general overview of the genetic causes of PD with mitochondrial involvement, whereas Fig. 4 depicts the proteins at their cellular action level.

**Table 1:** Nuclear genes linked to autosomal inherited PD with mitochondrial involvement

Protein	Function	Cellular localization	PD locus	Chromosomal location	Gene	Inheritance	Type of mutations	Key references
α-synuclein	Synaptic vesicle formation	Mainly cytoplasm	PARK1	4q21	*SNCA*	Dominant	Missense mutations (p.Ala30Pro, p.Glu46Lys, p.Ala53Thr), duplication, triplication	Chartier-Harlin *et al.* 2004; Ibanez *et al.* 2004; Kruger *et al.* 1998; Polymeropoulos *et al.* 1997; Singleton *et al.* 2003; Zarranz *et al.* 2004
Parkin	Ubiquitin E3 ligase	Mainly cytoplasm, OMM	PARK2	6q25.2–q27	*parkin*	Recessive	Missense, nonsense and frameshift mutations, intraexonic deletions or insertions, promoter and exon deletions, exon multiplications	Hedrich *et al.* 2004b; Kitada *et al.* 1998; Mata *et al.* 2004
Phosphatase and tensin homologue (PTEN)-induced kinase 1	Mitochondrial kinase	Mainly IMM, occasionally OMM	PARK6	1p36	*PINK1*	Recessive	Missense, nonsense and frameshift mutations, exon deletions	Li *et al.* 2005; Marongiu *et al.* 2007; Rogaeva *et al.* 2004; Valente *et al.* 2004
DJ-1	Redox sensor, mitochondrial antioxidant	Cytoplasm, IMS and matrix	PARK7	1p36.33–p36.12	*DJ-1*	Recessive	Missense and frameshift mutations, insertions and exon deletions	Abou-Sleiman *et al.* 2003; Bonifati *et al.* 2003; Hague *et al.* 2003
Leucine-rich repeat kinase 2	Multifunctional kinase/GTPase	Cytoplasm, OMM	PARK8	12q12	*LRRK2*	Dominant	Missense mutations	Berg *et al.* 2005; Paisan-Ruiz *et al.* 2004; Zimprich *et al.* 2004
High temperature requirement A2	Pro-apoptotic protease, important for mitochondrial homeostasis	IMS	PARK13	2p12	*HTRA2*	Dominant*	Missense mutations	Strauss *et al.* 2005

* In contrast with the other genes in the table, *HTRA2* was not identified through linkage studies, but was associated with PD using a candidate gene approach.

#### The inclusion protein SNCA

SNCA is the major constituent of Lewy bodies, the neuropathological hallmark of PD (Spillantini *et al.* 1997). *SNCA* is expressed throughout the brain (Solano *et al.* 2000) and is particularly enriched in presynaptic nerve terminals (Goedert 2001). It has potential roles in synaptic plasticity (George *et al.* 1995), regulation of dopamine neurotransmission (Abeliovich *et al.* 2000) and turnover of synaptic vesicles (Lotharius & Brundin 2002). In spite of extensive studies, the exact cellular function of SNCA remains unclear.

Missense mutations and multiplications of *SNCA* (4q21) cause autosomal dominant PD (Chartier-Harlin *et al.* 2004; Ibanez *et al.* 2004; Kruger *et al.* 1998; Polymeropoulos *et al.* 1997; Singleton *et al.* 2003; Zarranz *et al.* 2004), whereas association studies showed that increased *SNCA* expression because of variation in the *SNCA* promoter region conferred risk for sporadic PD (Maraganore *et al.* 2006; Pals *et al.* 2004).

Inducible expression of human p.Ala30Pro *SNCA* in PC12 cells (rat pheochromocytoma cells) decreased proteasome activity and increased sensitivity to mitochondria-dependent apoptosis (Tanaka *et al.* 2001). More recently human p.Ala53Thr *SNCA* overexpressing transgenic mice were reported to develop intraneuronal inclusions, as well as mtDNA damage and degeneration (Martin *et al.* 2006). Pathogenic p.Ala30Pro and p.Ala53Thr SNCA was also shown to be poorly degraded by chaperone-mediated autophagy (CMA) (Cuervo *et al.* 2004). Although these mutants bound with high affinity to the lysosomal membrane, they were not translocated into the lysosome. In addition to inhibiting their own degradation, they also blocked degradation of other CMA substrates; which is consistent with a toxic gain-of-function of these mutants.

On the other hand, loss of SNCA appears to have minimal effects because *SNCA* knockout mice were viable and fertile. Despite the lack of morphological abnormalities, these mice displayed a modest decrease in total striatal DA levels (Abeliovich *et al.* 2000), and a reduction in the reserve pool of synaptic vesicles in the hippocampus (Cabin *et al.* 2002).

#### The ubiquitin E3 ligase parkin

Ubiquitously expressed *parkin* encodes an ubiquitin E3 ligase (Shimura *et al.* 2000), which is a component of the UPS (Ciechanover 1998). Parkin was localized in mitochondria of proliferating cells and was also shown to play a role in mitochondrial biogenesis by regulating both transcription and replication of mtDNA (Kuroda *et al.* 2006).

Loss-of-function mutations in *parkin* cause autosomal recessive juvenile parkinsonism (Kitada *et al.* 1998). *Parkin* mutations may explain up to half of the patients with early onset PD and a family history compatible with recessive inheritance (Dekker *et al.* 2003). Mounting evidence suggests that heterozygous *parkin* mutations may increase susceptibility to late onset PD (Foroud *et al.* 2003; Oliveira *et al.* 2003; Sun *et al.* 2006; West *et al.* 2002). A promoter deletion in a family with a previously described heterozygous *parkin* exon 3-deletion recently extended the *parkin* mutation spectrum, as this compound heterozygous mutation was reported to result in complete absence of *parkin* expression (Lesage *et al.* 2007).

*Drosophila parkin* knockout mutants exhibited reduced lifespan, locomotor defects resulting from apoptotic muscle degeneration and male sterility. Mitochondrial structural alterations were prominent features of both muscle and germline pathology (Greene *et al.* 2003). Proteomics of midbrain from *parkin* knockout mice showed a decreased abundance of proteins involved in mitochondrial respiratory chain activity and protection against oxidative stress (Palacino *et al.* 2004).

*Parkin* overexpression models further support the role of parkin in mitochondria. *Parkin* overexpression in PC12 cells protected against ceramide-mediated cell death by delaying mitochondrial swelling, subsequent cytochrome *c* release and caspase 3 activation, and this protective effect was abrogated by *parkin* mutations (Darios *et al.* 2003). In human DAergic neuroblastoma cells (SH-SY5Y) *parkin* overexpression not only protected against apoptosis, but also decreased cellular ROS levels (Jiang *et al.* 2004).

On the other hand, mitochondrial dysfunction and oxidative stress can in turn affect parkin function, e.g. *S*-nitrosylation of parkin diminishes its ubiquitin E3 ligase activity and compromises its protective function (Chung *et al.* 2005). Such nitrosative stress combined with a single *parkin* mutation could lead to haploinsufficiency and might be particularly relevant for heterozygous *parkin* mutations associated with sporadic PD.

#### The mitochondrial kinase PINK1

The ubiquitously expressed PINK1 consists of an N-terminal mitochondrial targeting motif, a highly conserved serine–threonine kinase domain and a C-terminal autoregulatory domain (Silvestri *et al.* 2005). The PINK1 kinase is localized to the mitochondrial membranes (Gandhi *et al.* 2006).

Homozygous *PINK1* mutations were first described in three consanguineous families (Valente *et al.* 2004). *PINK1* loss-of-function mutations are estimated to account for 1–7% of early onset PD (Tan *et al.* 2006). Heterozygous *PINK1* mutations were also reported, and were threefold enriched in sporadic PD patients compared with control individuals (Abou-Sleiman *et al.* 2006). It is hypothesized that heterozygous *PINK1* mutations are a risk factor for the development of late onset PD (Khan *et al.* 2002).

Downregulation of *PINK1* expression by small interfering RNA (siRNA) decreased SH-SY5Y cell viability and increased apoptosis (Deng *et al.* 2005). RNA interference-mediated inactivation of the *PINK1* homologue in *Drosophila* resulted in progressive loss of DAergic neurons. This neurodegeneration was suppressed by human antioxidant superoxide dismutase (SOD) 1, which suggests that *PINK1* inactivation can induce neuronal death via an oxidative stress pathway (Wang *et al.* 2006). Notably, *PINK1* inactivation in *Drosophila* leads to a phenotype that shares marked similarity with that of *Drosophila parkin* knockout mutants, including shortened lifespan, apoptotic muscle degeneration, male sterility and defects in mitochondrial morphology. Transgenic expression of *parkin* markedly ameliorated all *PINK1* loss-of-function phenotypes, but not vice versa, suggesting that PINK1 and parkin function, at least in part, in the same pathway, with PINK1 functioning upstream of parkin (Clark *et al.* 2006; Park *et al.* 2006; Yang *et al.* 2006).

Overexpression of wild type *PINK1* in SH-SY5Y cells attenuated neuronal apoptosis by reducing the release of cytochrome *c* and subsequent activation of caspases under basal and apoptotic stress conditions. However, PD-related mutations and an artificial kinase-dead mutant abolished this protective effect (Petit *et al.* 2005). Most likely, the PINK1 kinase exerts its neuroprotective effect by phosphorylating specific mitochondrial proteins and in turn modulating their functions. However, the physiological substrates of PINK1 remain unknown.

#### The antioxidative DJ-1

*DJ-1* is expressed in a variety of tissues, including brain, and is partially localized to the mitochondrial matrix and IMS (Zhang *et al.* 2005a). DJ-1 is a H_2_O_2_ responsive protein, suggesting a function as antioxidant (Mitsumoto & Nakagawa 2001). DJ-1 acts as a redox sensor within cells and the acidification of a key Cys-residue (Cys106) seems to have an important signalling function. The same Cys-residue is also important for relocalization of DJ-1 to mitochondria (Canet-Aviles *et al.* 2004).

Recessively inherited *DJ-1* missense and exonic deletion mutations were first identified in two European early onset PD families (Bonifati *et al.* 2003). Additional loss-of-function mutations have since been identified (Abou-Sleiman *et al.* 2003, 2004; Hague *et al.* 2003; Hedrich *et al.* 2004a). Loss-of-function mutations in *DJ-1* are rare and are estimated to account for 1% of early onset PD (Lockhart *et al.* 2004). Single heterozygous *DJ-1* mutations were also found (Clark *et al.* 2004; Hedrich *et al.* 2004a), but as for heterozygous mutations in *parkin* and *PINK1*, their effect remains elusive.

*DJ-1* downregulation by siRNA in neuronal cell lines enhanced cell death by oxidative stress (Yokota *et al.* 2003). In *Drosophila* inhibition of *DJ-1α*, a *Drosophila* homologue of the human *DJ-1*, resulted in cellular accumulation of ROS, hypersensitivity to oxidative stress as well as dysfunction and degeneration of DAergic neurons (Yang *et al.* 2005). Knockout flies of *DJ-1α* and *DJ-1β*, the two *DJ-1* homologues in *Drosophila*, displayed a selective sensitivity to environmental toxins such as paraquat and rotenone (Meulener *et al.* 2005). DJ-1α and DJ-1β are prominently localized to enlarged and swollen mitochondria, implicating that the localization of DJ-1 to mitochondria is a protection mechanism against oxidative stress (Park *et al.* 2005). *DJ-1*-deficient mice show no gross anatomical or neuronal abnormalities and have normal numbers of SN DAergic neurons (Chen *et al.* 2005; Goldberg *et al.* 2005; Kim *et al.* 2005), however, their nigrostriatal pathway is dysfunctional (Chen *et al.* 2005; Goldberg *et al.* 2005), leading to higher dopamine concentrations and possibly more cellular oxidative stress.

#### The multidomain and multifunctional LRRK2

*LRRK2*, also known as *dardarin*, is expressed at low levels in most tissues (Paisan-Ruiz *et al.* 2004) and shows an expression pattern in brain that directly relates to the nigrostriatal dopamine system. It is expressed in the dopamine target areas, striatum and frontal cortex, whereas the dopamine neurons themselves are devoid of *LRRK2* messenger RNA (Galter *et al.* 2006). On a cellular level LRRK2 resides diffusely throughout the cytosol, but also associates with the OMM (West *et al.* 2005). Sequence analysis indicates that LRRK2 comprises several domains including a leucine-rich repeat domain, a GTPase (guanosine triphosphatase) domain Ras of complex proteins (Roc) followed by its associated C-terminal of Roc domain, a MLK-like domain and a C-terminal WD40 domain (Paisan-Ruiz *et al.* 2004; Zimprich *et al.* 2004). The kinase activity may be the link between LRRK2 and its role in PD pathogenesis.

Mutations in *LRRK2* cause autosomal dominant late onset PD (Paisan-Ruiz *et al.* 2004; Zimprich *et al.* 2004). *LRRK2* missense mutations reported so far are distributed along the protein (Berg *et al.* 2005; Khan *et al.* 2005; Paisan-Ruiz *et al.* 2004; Zimprich *et al.* 2004). p.Gly2019Ser is the most common pathogenic *LRRK2* mutation in Caucasians and accounts for approximately 5% of familial PD and 1.5% of sporadic PD (Di Fonzo *et al.* 2005; Gilks *et al.* 2005; Nichols *et al.* 2005). We recently detected a founder effect of p.Arg1441Cys, the second most frequent pathogenic *LRRK2* mutation, in the Belgian population (Nuytemans *et al.* in preparation).

p.Arg1441Cys in the GTPase domain and p.Gly2019Ser in the MLK-like domain were shown to result in increased kinase activity *in vitro* (West *et al.* 2005), a similar increase was seen for p.Ile2020Thr (Gloeckner *et al.* 2006). These findings are in favour of a gain-of-function mechanism for *LRRK2*-linked disease. Expression of p.Arg1441Cys and p.Gly2019Ser *LRRK2* caused neuronal degeneration in both SH-SY5Y cells and mouse primary neurons (Smith *et al.* 2005). Overexpression of p.Gly2019Ser *LRRK2* in rats did not alter normal appearance of DAergic neurons, but significantly increased apoptosis (MacLeod *et al.* 2006). Extensive *in vivo* validation of these data is, however, required.

#### The mitochondrial serine protease HTRA2

HTRA2 is ubiquitously expressed (Faccio *et al.* 2000) and nuclear encoded but carries a mitochondrial targeting sequence at its N-terminus. HTRA2 has an N-terminal IAP-binding motif, a serine protease domain and, at its C-terminus, a PDZ domain, which restricts access to the active site of the serine protease (Li *et al.* 2002; Verhagen *et al.* 2002). HTRA2 is localized to the IMS, but is released into the cytosol following apoptotic stimuli. In the cytosol it can induce apoptosis in a caspase-dependent manner by antagonizing caspase–IAP interaction, or in a caspase-independent manner relying on its serine protease activity (Hegde *et al.* 2002).

Transient *HTRA2* overexpression in HEK293 cells (human embryonic kidney cells) induced apoptosis even in the absence of an apoptotic insult, whereas artificial protease-inactive mutant HTRA2 (Li *et al.* 2002) had lower death-inducing ability (Martins *et al.* 2002). In non-apoptotic conditions the proteolytic activity of HTRA2 is involved in maintaining mitochondrial homeostasis, as was first shown in the *mnd2* mouse for motor neuron degeneration 2 (Jones *et al.* 2003) and later also in *HTRA2* knockout mice (Martins *et al.* 2004). Both *mnd2* and *HTRA2* knockout mice died prematurely because of loss of striatal neurons. The identification of a p.Gly399Ser mutation in the HTRA2 PDZ domain in patients with sporadic PD (Strauss *et al.* 2005) was of direct relevance to PD. At the cellular level p.Gly399Ser HTRA2 impaired the regulation of proteolytic activity, caused mitochondrial swelling, decreased mitochondrial membrane potential and increased staurosporine-induced cell death. We recently identified another PD-specific HTRA2 mutation in the PDZ domain, p.Arg404Trp, in an extensive mutation analysis of over 250 Belgian PD patients (Bogaerts *et al.* unpublished data).

#### Other genes relevant to mitochondrial function and oxidative stress

Several polymorphisms in functional candidate genes related to mitochondrial function and oxidative stress have been studied for their possible association with PD. MAO, a principal enzyme of dopamine metabolism that has two isoforms: MAO-A and MAO-B, was tested for its supposed role in PD pathogenesis: a polymorphism in intron 13 of MAO-B was significantly associated with increased risk of PD (Kang *et al.* 2006; Tan *et al.* 2000b). Another polymorphism in exon 22 of inducible nitric oxide synthase (encoded by *NOS2A)*, an enzyme that produces NO (Fig. 2), showed an inverse association with PD (Hague *et al.* 2004; Levecque *et al.* 2003). Although NO is a biological messenger, NO overproduction contributes to oxidative stress, impairment of mitochondria and overload of degradation pathways. Interestingly *NOS2A* polymorphisms were also reported to interact with smoking (Hancock *et al.* 2006). Glutathione *S*-transferase (GST) variants have been extensively studied in PD because of their ability to detoxify exogenous and endogenous toxic substances. Moreover, polymorphisms in *GST Omega-1 and -2* were shown to modify age at onset of PD (Li *et al.* 2006). These genes represent only a handful of the list of functional candidate genes for PD, but their implication in PD at least supports the pathophysiological significance of mitochondrial dysfunction to PD.

#### The relevance of genetic findings in PD

The future of drug therapies depends on the development of animal models that strongly recapitulate the disease, not only to discover the mechanisms of disease but also to identify new neuroprotective drugs and to test their efficacy. The genetic findings described above provide a direct mechanistic link with PD and therefore animal models expressing the discovered PD-causing mutations probably mimic human PD better than neurotoxic animal models. The identified PD genes themselves represent direct targets for therapeutic application, e.g. by RNA interference. In addition, evidence from genetic studies indirectly suggests that mitochondrial dysfunction plays an integrative role in PD pathogenesis, and therefore mitochondrial function and mechanisms to cell death represent valid targets for potential neuroprotective therapies.

## Direct relevance of genetic research to treatment

### Transgenic mouse models of PD

The identification of causal PD genes in rare Mendelian forms of PD triggered the development of transgenic mouse models. The underlying hypothesis is that mutations causing Mendelian forms of PD highlight pathways, relevant to sporadic PD (Melrose *et al.* 2006). However all of these mouse models had shortcomings, of which the most notable is failure to exhibit degeneration of the nigrostriatal system, a key pathological feature of PD (Manning-Bog & Langston 2007). Efforts to create genetic models of PD in simple systems, e.g. *Drosophila* and yeast, have resulted in robust phenotypes. However, the principal value of these simple models of PD is perhaps that results in these systems might be used to improve current mouse models of PD (Whitworth *et al.* 2006).

Given its clear link with PD, a variety of *SNCA* transgenic mice have been generated, varying in tissue specificity, mutation and expression level (for a review see Fernagut & Chesselet 2004). The developed *SNCA* transgenic models proved to be valuable in partially recapitulating the predisposition of certain neuronal populations to SNCA aggregation. *Parkin* knockout mice had normal numbers of DAergic neurons, but displayed subtle deficits in behaviour and DAergic neurotransmission (Goldberg *et al.* 2003; Itier *et al.* 2003). Similar to *parkin* knockouts, loss of *DJ-1* did not result in loss of SN DAergic neurons (Chen *et al.* 2005; Goldberg *et al.* 2005; Kim *et al.* 2005), but again subtle behavioural deficits and increased striatal DA reuptake were reported. So far *PINK1* knockout mice have not been reported, but silencing of *PINK1* expression in mice by use of RNA interference did not cause a loss of DAergic neurons either (Zhou *et al.* 2007). *LRRK2* transgenic mice have also not been reported. It is hoped that these transgenic mice display a more robust pathological phenotype (Melrose *et al.* 2006). However, considerable variability in pathological phenotype was already documented for several pathogenic *LRRK2* mutations (Rajput *et al.* 2006; Zimprich *et al.* 2004).

Yet no perfect animal model for PD exists; most transgenic mouse lines do not show damage to the nigrostriatal system, but rather have subtle changes. Opposed to this, neurotoxic animal models do show degeneration of SN DAergic neurons. Combining these animal models might thus be very fruitful to develop new and better PD models. Such fused genetic and neurotoxic models might better capture the full spectrum of PD features, and provide excellent means to study gene–environment interactions (Manning-Bog & Langston 2007). Nevertheless, genetic mouse models have at least illustrated that neuronal dysfunction in PD most likely occurs long before cell loss. Current transgenic or knockout mouse models might indeed provide insights into the early stages of PD (Fleming *et al.* 2005), allowing us to decipher pathological processes that take place in brain exposed to a PD-causing insult before degeneration of the nigrostriatal system occurs, or before the disease begins elsewhere (Litvan *et al.* 2007).

### Gene-based therapy

A straightforward approach, supported by genetic findings, is the possibility to lower SNCA levels. Indeed, given the gain-of-function mechanism of PD-linked SNCA mutations, silencing expression of mutant *SNCA* or reducing overexpression of wild-type *SNCA* is an attractive target for therapeutic RNA interference (Gonzalez-Alegre 2007). Allele-specific silencing of mutant *SNCA* was already shown *in vitro*, as well as suppression of overexpressed human *SNCA in vivo* in rat brain (Sapru *et al.* 2006). However, these studies need replication in different animal models of PD. A vaccination approach was also tested: immunization of human *SNCA* transgenic mice with recombinant human SNCA led to decreased SNCA accumulation in neuronal cell bodies and synapses, as well as reduced neurodegeneration (Masliah *et al.* 2005). Human trials on the proposed vaccine are currently underway (Singh *et al.* 2007). For *parkin*, *PINK1* and *DJ-1*, where PD is caused by loss-of-function mechanisms, increasing expression is likely to ameliorate the phenotype (Farrer 2006). For example, *parkin* gene therapy was already shown to be effective against *SNCA* overexpression in rats (Mochizuki *et al.* 2006). Last but not least, the identification of *LRRK2* mutations as a frequent cause of familial and sporadic PD was a landmark discovery in PD research. The increased kinase activity observed for mutant LRRK2 (West *et al.* 2005) may, if proved *in vivo*, translate relatively quickly into a novel treatment option involving kinase inhibition (Litvan *et al.* 2007). However, the physiological targets for LRRK2 activity are not yet known, hence uncertainty remains whether we can target this kinase activity without detrimental effects.

## Drugs targeting mitochondrial pathways to PD

A scientific rationale, evidence of blood brain barrier (BBB) penetration, adequate safety data, efficacy in animal models and/or preliminary efficacy data in humans are requirements to identify promising neuroprotective drugs in PD (Ravina *et al.* 2003). In contrast with symptomatic treatments, neuroprotective drugs aim to slow or halt the progression of PD by a direct effect on the vulnerable cells (Olanow & Jankovic 2005).

**Figure 5:**
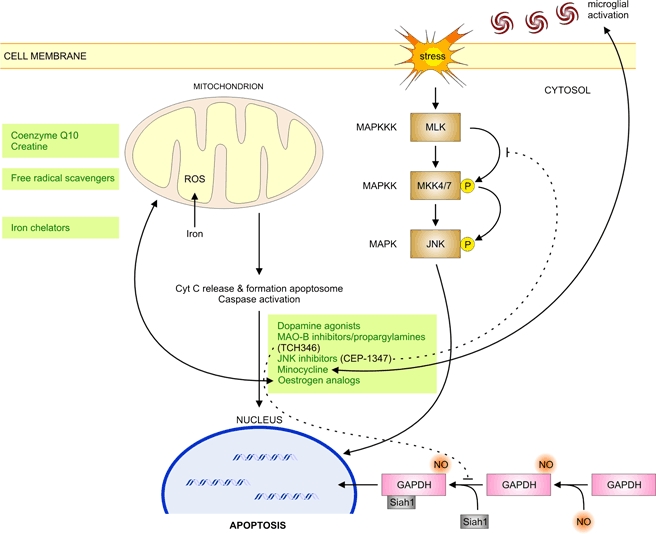
**Entry points for PD therapy involving mitochondria.** Green boxes highlight potential neuroprotective drugs at their respective action levels, and dotted lines indicate premature termination of clinical trials for promising neuroprotective drugs. Abbreviations: Cyt C, cytochrome *c*; MAPKK, MAPK kinase; MAPKKK, MAPKK kinase; MKK4/7, MAPK kinases 4/7; P, phosphorylated.

The neuroprotective drugs include dopamine agonists and MAO-B inhibitors, which are already used for years to improve symptoms in the early stages of PD. Based on our current understanding of PD pathogenesis and mitochondria-related mechanisms of cell death, neuroprotective drugs can be divided into drugs that (1) attenuate mitochondrial apoptosis, (2) reduce oxidative stress in mitochondria or (3) directly target mitochondrial function (Fig. 5).

### Antiapoptotic

#### Dopamine agonists

Dopamine agonists have proven efficacy in the early stages of PD. Dopamine agonists directly stimulate dopamine receptors and unlike levodopa their symptomatic effect does not depend on the presence of dopamine producing neurons (Junghanns *et al.* 2004). The neuroprotective effect of dopamine agonists may be the result of different mechanisms, including a ‘levodopa sparing’ effect (delaying the need for levodopa therapy) (Holloway *et al.* 2000; Rascol *et al.* 2000), antioxidative properties by scavenging free radicals (Iida *et al.* 1999), as well as inhibition of apoptosis (Nair *et al.* 2003).

Pramipexole is perhaps the best-studied dopamine agonist. Pramipexole decreased apoptotic cell death in SH-SY5Y cells exposed to MPP^+^ and rotenone (Kitamura *et al.* 1998; Schapira *et al.* 2002) and was shown to inhibit opening of the PT pore, reduce release of cytochrome *c* and decrease activation of caspase 3 (Cassarino *et al.* 1998; Gu *et al.* 2004; King *et al.* 2001). *In vivo* studies showed that marmosets (non-human primates) pretreated with pramipexole had significantly greater numbers of surviving SNpc neurons upon MPTP treatment (Iravani *et al.* 2006). The need for pretreatment, which is in agreement with earlier observations in SH-SY5Y cells (Gu *et al.* 2004; Kitamura *et al.* 1998), does not preclude clinical application of pramipexole, but rather emphasizes the importance of pramipexole administration early in the disease process to protect the remaining SN neurons from future dysfunction or degeneration.

Two prospective randomized double-blind clinical trials in early PD patients (diagnosed within 5 years) supported the neuroprotective effect of dopamine agonists (Schapira 2003), but further trials are needed to confirm their neuroprotective potential.

#### MAO-B inhibitors and propargylamines

MAO-A and MAO-B are tightly associated with the OMM and oxidatively deaminate monoamine neurotransmitters (e.g. dopamine), as well as exogenous monoamines (e.g. tyramine). MAO inhibitors were considered in PD therapy as dopamine sparing drugs, however, the first generation of non-selective MAO inhibitors had a major side-effect, known as the ‘cheese reaction’ (hypertensive crisis because of potentiation of the sympathomimetic activity of tyramine by peripheral inhibition of its MAO metabolism). Selective MAO-B inhibitors, such as the propargylamine-related l-deprenyl (Birkmayer *et al.* 1975) and the more potent rasagiline (Siderowf & Stern 2006), do not have such effect (Youdim & Weinstock 2004). Neuroprotection by l-deprenyl (Birkmayer *et al.* 1985) and other propargylamines is most likely because of interference with apoptotic signalling pathways rather than by MAO-B inhibition itself (Tatton *et al.* 2003a). In addition, l-deprenyl was shown to inhibit the formation of SNCA fibrils and to destabilize preformed SNCA fibrils (Ono *et al.* 2007).

In SH-SY5Y cells rasagiline suppressed apoptosis by preventing the fall in ΔΨ_m_ and opening of the mitochondrial PT pore, preventing nuclear translocation of GAPDH (Youdim & Weinstock 2001) and increasing expression of antiapoptotic Bcl-2 family members (Akao *et al.* 2002). In rats and marmosets l-deprenyl and rasagiline attenuated the neurotoxic effects of MPTP (Kupsch *et al.* 2001; Wu *et al.* 2000).

The neuroprotective effect of l-deprenyl and rasagiline was tested in prospective randomized multicentre placebo-controlled studies: the DATATOP (Deprenyl and Tocopherol Antioxidative Therapy for Parkinson’s disease) (Shoulson *et al.* 1993) and TEMPO study (TVP-1012 (rasagiline mesylate) in Early Monotherapy for Parkinson’s disease Outpatients) (Parkinson Study Group 2004a). The TEMPO study showed that PD patients treated with rasagiline for 12 months showed less functional decline than patients whose treatment was delayed for 6 months. Such difference cannot be simply explained by a symptomatic effect, which was a confounder in studies of l-deprenyl (Olanow *et al.* 1995; Shoulson *et al.* 2002), and is consistent with rasagiline having a neuroprotective effect. A larger double-blind delayed-start study is under way to confirm these data.

Another promising drug with potential neuroprotective effect is TCH346 (also known as CGP3466). TCH346 incorporates a propargyl ring within its molecular structure and resembles l-deprenyl, but does not inhibit MAO-B and was therefore not anticipated to have confounding symptomatic effects (Olanow *et al.* 2006).

TCH346 prevented nuclear translocation of GAPDH in a cellular system (Hara *et al.* 2006), and in bilaterally MPTP-treated monkeys TCH346 prevented death of SN DAergic neurons (Andringa *et al.* 2003). Preclinical safety studies and small trials in healthy volunteers showed that TCH346 was well tolerated and free of clinically significant adverse effects.

A highly anticipated first double-blind placebo-controlled clinical trial was already completed (Olanow *et al.* 2006). Unfortunately, no significant differences were found between any of the tested doses and placebo with respect to the primary outcome (time to disability requiring DAergic treatment) or secondary outcome measures [e.g. changes in Unified Parkinson’s Disease Rating Scale (UPDRS)] in patients with early untreated PD.

Nevertheless, the evidence for neuroprotection by l-deprenyl and nasagiline still deserves further study. Both propargylamines differ from TCH346 by their additional MAO-B inhibitory effects, which might not be redundant to explain the apparent differences in their potential neuroprotective effects.

#### JNK inhibitors

The first successful compound targeting the JNK signalling pathway was CEP-1347 (Maroney *et al.* 1998). CEP-1347 does not directly inhibit JNK, but reduces neuronal cell death by MLK inhibition (Maroney *et al.* 2001). JNK activation is relevant to PD pathogenesis, as activated JNK was associated with SNCA pathology in *SNCA* p.Ala30Pro transgenic mice (Frasier *et al.* 2005) and loss of parkin function was already shown to activate the JNK pathway in *Drosophila* (Cha *et al.* 2005).

CEP-1347 attenuated the neurotoxic effects of MPP^+^ in differentiated SH-SY5Y cells (Mathiasen *et al.* 2004), and reduced MPTP-mediated nigrostriatal DAergic neuron loss *in vivo* in mice (Saporito *et al.* 1999). A placebo-controlled pilot study showed that CEP-1347 was safe and well tolerated by PD patients (Parkinson Study Group 2004b), and this supported the initiation of a large trial of longer duration with CEP-1347: the PRECEPT study (Parkinson Research Examination of CEP-1347 Trial).

The PRECEPT study was, however, prematurely abrogated when an interim analysis showed that it would be futile to continue experimental treatment. In all tested CEP-1347 dose regimens more patients had reached the primary and secondary end-points compared with patients randomized to placebo (Shoulson *et al.* 2006). Again these findings contrasted with research in preclinical models that predicted favourable effects of CEP-1347 on the progression of PD.

#### Minocycline

Minocycline, a second-generation tetracycline, has a proven safety record in humans. Among several activities minocycline impairs microglial activation, neuroinflammation and apoptosis (Yong *et al.* 2004). In midbrain cultures of *parkin* knockout mice minocycline neuroprotection was related to inactivation of microglial cells and protection against neuronal apoptotic cell death (Casarejos *et al.* 2006). Increasing evidence suggests that its antiapoptotic effect is achieved through mechanisms acting at the level of mitochondria.

Minocycline inhibited mitochondrial PT mediated cytochrome *c* release in isolated brain mitochondria (Zhu *et al.* 2002), and in kidney cells minocycline not only induced antiapoptotic Bcl-2, but also antagonized pro-apoptotic Bcl-2 family members (Wang *et al.* 2004). Furthermore, minocycline was shown to prevent nigrostriatal DAergic neurodegeneration in the MPTP mouse model of PD (Du *et al.* 2001; Wu *et al.* 2002). In contrast with numerous reports of beneficial effects of minocycline, some studies reported significant exacerbation of MPTP-induced damage by minocycline in mice (Yang *et al.* 2003) and monkeys (Diguet *et al.* 2004). It remains, however, plausible that minocycline has different, sometimes even deleterious, effects according to its mode of administration and dose. A recent study suggested that the mitochondrial effects of minocycline contribute to toxicity rather than tissue protection at high dosing in animals and humans (Mansson *et al.* 2007). The possible harmful effects of minocycline must thus be ceaselessly kept in mind when considering minocycline use in clinical PD settings.

A randomized, double-blind, futility clinical trial of minocycline in early PD patients could not reject minocycline as futile (Ravina *et al.* 2006). Such futility trials are intended to determine if treatments are worthy of larger and longer term studies, or if they should be abandoned (Elm *et al.* 2005). Although the results of this futility study do not provide evidence for clinical use of minocycline in the treatment of PD, they indicate that minocycline at least merits further consideration for larger clinical trials.

#### Oestrogen analogues

Postmenopausal oestrogen replacement therapy in women with early PD was positively associated with lower symptom severity (Saunders-Pullman *et al.* 1999) and suggested a potential neuroprotective effect of oestrogens. Indeed, numerous *in vitro* and *in vivo* studies established that oestrogens act as neuroprotectants when challenged by various toxic stresses.

Several effects might explain the beneficial actions of oestrogens on brain; including anti-inflammatory activity and protection against apoptosis (Maggi *et al.* 2004). These effects appear to be primarily mediated through activation of intracellular oestrogen receptors (ER) and result in modulated transcription of oestrogen target genes, e.g. upregulated expression of antiapoptotic Bcl-2 (Garcia-Segura *et al.* 1998). Neuroprotective effects of oestrogens might also result from ER-independent mechanisms, including antioxidant effects (Moosmann & Behl 1999) related to their basic chemical properties as hydrophobic phenolic molecules. Mechanistic and structure–activity studies with oestrogens yielded a model in which they intercalate into cell membranes where they block lipid peroxidation reactions, and thereby stabilize membrane structure (Behl *et al.* 1997), which is especially critical to mitochondrial function.

Both the naturally occurring 17β-oestradiol (17β-E2) and its 17α-oestradiol (17α-E2) isomer, which is at least 200-fold less active as transactivating hormone, protected DAergic neurons from MPP^+^-induced neuronal death (Sawada *et al.* 2002). 17β-E2 showed beneficial effects in animal models of PD using MPTP as lesioning agent (Callier *et al.* 2000; Ramirez *et al.* 2003), and 17α-E2 treatment in 6-hydroxydopamine injected rats later showed significantly improved behavioural outcomes (Dykens *et al.* 2005b).

A first small double-blind placebo-controlled clinical trial of 17α-E2 was completed including eight healthy postmenopausal women; the tested doses of 17α-E2 were well tolerated and no adverse events were reported (Dykens *et al.* 2005a). However, the neuroprotective, or better mitoprotective, effects of 17α-E2 remain to be tested in PD patients.

### Antioxidants

#### Iron chelators

Alterations in cellular iron metabolism and iron-induced oxidative stress are important factors in the pathogenesis of PD (Bharath *et al.* 2002; Riederer *et al.* 1989; Sofic *et al.* 1991; Youdim *et al.* 1993). Moreover, iron was shown to accelerate the aggregation of SNCA (Kalivendi *et al.* 2004) and to induce alterations of parkin solubility, with depletion of soluble functional parkin leading to reduced proteasomal activities and increased cell death (Wang *et al.* 2005). Reducing the SN iron content through iron chelation might thus be a straightforward therapeutic approach to the treatment of PD (Zhang *et al.* 2005b).

Desferrioxamine, the prototype of iron chelators (Jiang *et al.* 2006), blocked iron-induced oxidative damage in SK-N-SH neuroblastoma cells (DAergic origin) (Sangchot *et al.* 2002). In rat brain mitochondria desferrioxamine also protected mitochondrial complex I activity against inhibition by 6-hydroxydopamine, possibly through a combined neuroprotective effect of iron chelation and NADH dehydrogenase activation (Glinka *et al.* 1996, 1998). The neuroprotective effect of preinjected desferrioxamine was later confirmed in rat (Ben Shachar *et al.* 1991; Youdim *et al.* 2004) and mice models (Lan & Jiang 1997).

As desferrioxamine has poor BBB penetration, other lipophilic chelators with enhanced BBB penetration have been synthesized; e.g. VK-28 (Ben Shachar *et al.* 2004). Novel iron chelators were further developed based on VK-28, that combine its iron chelating potency with a MAO inhibitory propargylamine moiety (Youdim *et al.* 2005), e.g. the multifunctional M30. M30 exhibited neuroprotective activity upon *in vitro* and *in vivo* testing (Gal *et al.* 2005; Zheng *et al.* 2005), but its effect in PD patients remains to be evaluated. Nevertheless, as multifunctional compounds possess various targets within the central nervous system, they may provide better efficacy and utility as potential disease-modifying drugs (Youdim & Buccafusco 2005).

#### Free radical scavengers

Under physiological conditions several cellular mechanisms are able to neutralize a possible excess of free radicals and thereby prevent oxidative stress. These comprise free radical scavengers, which are either endogenously produced molecules or nutrients, and scavenger enzymes such as SOD, glutathione peroxidase and catalase (Fig. 2). The free radical scavengers include vitamins A, C and E, as well as glutathione in its reduced form, and melatonin (Contestabile 2001). RNA interference-mediated inactivation of the *Drosophila PINK1* homologue already showed that addition of SOD1 and vitamin E to the flies’ diet inhibited degeneration of photoreceptor neurons (Wang *et al.* 2006).

The lipid-soluble vitamin E received much attention as a chain-breaking antioxidant that prevents propagation of the radical chain (Burton *et al.* 1983). *In vitro* studies indicated oxidation of vitamin E upon incubation with free radicals in rat brain mitochondria (Vatassery *et al.* 1995) and protection against H_2_O_2_-induced oxidative stress in a murine hippocampal-derived cell line (Behl 2000). However *in vivo* studies did not consistently find positive effects of vitamin E administration (Gong *et al.* 1991; Roghani & Behzadi 2001).

Already in 1987 α-tocopherol, the biologically most active form of vitamin E, was selected for the first clinical trial of neuroprotection in PD: the DATATOP study (Shoulson *et al.* 1993). This study did not show beneficial effects of supplemental α-tocopherol intake in PD patients, but a more recent meta-analysis suggested that diets rich in vitamine E might exert a protective effect against the development of PD (Etminan *et al.* 2005). There is, however, some concern that high-dosage vitamin E supplementation may be harmful (Miller *et al.* 2005).

Use of reduced gluthathione as a free radical scavenger is hampered by a poor BBB permeability (Zeevalk *et al.* 2007). On the other hand, the pineal hormone melatonin is a highly lipophilic free radical scavenger (Reiter *et al.* 1993) and antioxidant (Tan *et al.* 2000a). Some of the melatonin metabolites resulting from scavenging actions are also efficient free radical scavengers (Lopez-Burillo *et al.* 2003). The action of melatonin as a free radical scavenger is thus a sequence of scavenging reactions, resulting in a cascade of protective reactions.

Pharmacological doses of melatonin, which greatly exceed endogenous levels, were shown to be neuroprotective in different neurotoxic models of PD (Joo *et al.* 1998; Thomas & Mohanakumar 2004), but to our knowledge melatonin has not yet been tested for the treatment of PD in clinical trials.

### Targeting mitochondrial function

#### Coenzyme Q_10_

Coenzyme Q shuttles electrons from complexes I/II to complex III of the electron transport chain, and the predominant form of coenzyme Q in humans is coenzyme Q_10_. In addition, ubiquinol (its reduced form) functions as antioxidant, prevents lipid peroxidation in most subcellular membranes and also protects mitochondrial membrane proteins and DNA from ROS-induced oxidative damage (Ernster & Dallner 1995).

Coenzyme Q_10_ was shown to prevent *in vitro* mitochondrial PT pore opening upon apoptotic stimuli (Papucci *et al.* 2003). In aged mice coenzyme Q_10_ attenuated MPTP-induced loss of striatal dopamine and DAergic axons (Beal *et al.* 1998). In addition, PD patients showed lower levels of coenzyme Q_10_ in platelet mitochondria (Shults *et al.* 1997), as well as elevated levels of oxidized coenzyme Q_10_ (Sohmiya *et al.* 2004) compared with control individuals.

A pilot study in PD patients showed that oral administration of coenzyme Q_10_ was well tolerated and caused a trend towards increased complex I activity (Shults *et al.* 1998). A later study also showed reduced functional decline in PD patients taking coenzyme Q_10_ (Shults *et al.* 2002). More recently, a randomized, double-blind, futility clinical trial of coenzyme Q_10_ in early PD could not reject coenzyme Q_10_ as futile (The NINDS NET-PD Investigators 2007). However, large trials are still needed to evaluate the long term effects of coenzyme Q_10_ intake and to assess its disease-modifying potential in the treatment of PD patients.

#### Creatine

The endogenous guanidine-derived creatine is a substrate for cytosolic and mitochondrial creatine kinases (CK). Aerobic glycolysis, coupled to mitochondrial ATP synthesis via oxidative phosphorylation, is the primary pathway of ATP synthesis in brain, but in addition the CK/phosphocreatine system provides a rapid alternative source for ATP synthesis (Wallimann *et al.* 1992). Mitochondrial CK together with high cytoplasmic creatine levels also inhibits mitochondrial PT and the consequent triggering of apoptosis (Andres *et al.* 2005).

In primary neuronal cultures creatine exerted neuroprotective effects against neurotoxic insults as MPP^+^ exposure (Andres *et al.* 2005). Oral supplementation with creatine protected against MPTP-induced dopamine depletion in mice and reduced damage to the nigrostriatal DAergic system (Matthews *et al.* 1999).

A double-blind placebo-controlled pilot study on the effect of oral creatine supplementation on PD progression did not find a treatment effect on UPDRS scores, although a significantly smaller dose increase of DAergic therapy in creatine-treated patients indicated a potential neuroprotective effect (Bender *et al.* 2006a). A randomized, double-blind, futility clinical trial of creatine in early PD could not reject creatine as futile (Ravina *et al.* 2006). Creatine thus remains a promising neuroprotective drug for PD, but its neuroprotective potential may only be shown by large multicentre studies over an extended observation period.

## Conclusion

Molecular genetic studies identified key proteins involved in PD pathogenesis. Mutations or polymorphisms in mtDNA, but especially mutations in the nuclear-encoded *SNCA*, *parkin*, *PINK1*, *DJ-1*, *LRRK2* and *HTRA2* provided direct or indirect links between mitochondria and PD pathogenesis. Other chromosomal loci still await refinement and characterization (Bogaerts *et al.* 2007; Gasser *et al.* 1998; Hicks *et al.* 2002; Pankratz *et al.* 2003) and will further increase our knowledge of the basic mechanisms underlying PD pathogenesis.

Perhaps the most relevant question for research into the causes of PD is: how can we exploit the gained knowledge on mitochondrial involvement in PD to develop better therapies? Neuroprotective therapies that slow or halt disease progression remain an unmet need. Our insights into the pathogenic mechanisms of cell death in PD led to several potential neuroprotective drugs, which have actions as attenuating mitochondrial apoptosis and reducing oxidative stress in mitochondria, or directly target mitochondrial function. The selection of drugs covered in this review all exert at least one of the three above mentioned actions, along with possible involvements in other non-mitochondrial pathways to PD.

However, no drug has yet been shown to be neuroprotective in PD patients, although several were tested in clinical trials. This discrepancy, reported for several potential neuroprotective drugs targeting different elements of the mitochondrial pathway, raises serious concerns as to the suitability of currently available models (Waldmeier *et al.* 2006). Particularly, the currently used animal models, typically acute or subacute neurotoxic models, might not adequately recapitulate the pathogenesis of the slower progressive neurodegenerative process in humans. Therefore, genetic models of PD are highly desirable because they produce a delayed onset and a neurodegenerative process that probably mimics human PD better than neurotoxic models.

On the other hand, the clinical end-points used to assess neuroprotective drugs in PD, such as need for symptomatic treatment or change in UPDRS score, may be confounded by symptomatic effects, or may simply be too insensitive. True biomarkers that accurately reflect disease progression remain an urgent priority. An additional concern is that DA neurons may already be beyond rescue when clinical features appear. This reinforces the need for biomarkers to identify patients who are in the presymptomatic or early stages of PD. Ideally, neuroprotective drugs should be administered during this early period (Halbig *et al.* 2004).

Last but not least a very important questions remains: do we target the right pathway? Moreover, how can we fit all the cellular processes uncovered by genes involved in familial forms of PD in the mitochondrial pathway? And is this mitochondrial pathway also relevant to sporadic forms of PD? Association studies in sporadic PD patients, showing modest effects for candidate genes involved in mitochondrial function and oxidative stress, support this contention. On the other hand, monogenic PD explains only 20% of early onset PD and less than 3% of late onset PD at best (Klein 2006). In most PD patients, the loss of DAergic neurons results from a combination of exogenous stresses and a genetic predisposition. Given this complexity, it is unlikely that any neuroprotective drug will be applicable to the full spectrum of PD patients. However, it is more plausible that mixtures of therapies targeting several different processes converging in a common pathway may prove useful in PD neuroprotection. The recent development of multifunctional compounds represents a move into that direction.

As the repeated failure of neuroprotective drugs in clinical trials has unfortunately shown, we must acknowledge that at present we merely have a rough sketch of the mitochondrial pathway to neurodegeneration in PD. This sketch turns out inadequate to predict fruitful neuroprotective approaches and further research is definitely needed. Nevertheless, the future looks bright. Our understanding of PD pathogenesis is rapidly evolving, boosted by a massive amount of research into the genetic causes of PD, the influence of genetic and environmental susceptibility factors on disease penetrance and manifestation, and the development of better models to test potential neuroprotective therapies. New insights from these future studies will (re)direct our efforts and eventually will provide us with neuroprotective therapies that might really make the change in the treatment of PD.

## Glossary

Association study: Study of the correlation between a genetic variant in a population and a disease. If a correlation is observed, then there is said to be an association between the variant and the disease.

Candidate gene: Gene with evidence for a possible role in the disease under study.

DAergic: Related to or activated by dopamine.

Early PD: Early stages of PD with subtle impairment. In clinical trials, early PD patients have Hoehn and Yahr stage I (unilateral disease) or stage II (bilateral disease with preservation of postural reflexes) PD for less than 5 years. The Hoehn and Yahr rating scale is a simple and popular scale to establish the severity of PD with stages ranging from I to V (wheelchair-bound or walking only with assistance).

Early onset PD: Having an onset at or before 45 years of age.

Haploinsufficiency: Situation in which half of the normal level of gene expression is not sufficient to permit cells to function normally.

Haplotype: Sequential set of alleles present on the same chromosome.

Linkage analysis: Mapping loci by genotyping genetic markers in families to identify chromosome regions implicated in disease. Such linked regions are of great help in the identification of genetic variants causing disease.

Meta-analysis: A statistical analysis combining the results of several independent studies.

Mitochondrial respiratory chain (electron transport chain): Collective term used to describe the peptides, organized in five enzymatic mitochondrial complexes (I–V), and the electron shuttle molecules that are needed to produce ATP.

Oxidative phosphorylation: Phosphorylation of adenosine 5′-diphosphate to ATP driven by the transfer of electrons to oxygen.

SH-SY5Y: Human neuroblastoma subclonal cell line of the neuroepithelioma cell line SK-N-SH. These cells are widely used in studies addressing the molecular mechanisms of neurodegenerative disease.

UPS: Cellular system responsible for the degradation of damaged or misfolded proteins. Ubiquitin molecules are attached to lysine residues of a given protein and target the protein for destruction by the multienzyme proteasome.

UPDRS: Unified Parkinson’s Disease Rating Scale used to monitor the progression of PD. It is the most widely used clinical rating scale for PD and consists of a four-part evaluation: I non-motor experiences of daily living, II motor experiences of daily living, III motor examination and IV motor complications.
